# Histone Lysine Methylation in Diabetic Nephropathy

**DOI:** 10.1155/2014/654148

**Published:** 2014-08-25

**Authors:** Guang-dong Sun, Wen-peng Cui, Qiao-yan Guo, Li-ning Miao

**Affiliations:** Department of Nephrology, Second Hospital of Jilin University, Changchun 130041, China

## Abstract

Diabetic nephropathy (DN) belongs to debilitating microvascular complications of diabetes and is the leading cause of end-stage renal diseases worldwide. Furthermore, outcomes from the DCCT/EDIC study showed that DN often persists and progresses despite intensive glucose control in many diabetes patients, possibly as a result of prior episode of hyperglycemia, which is called “metabolic memory.” The underlying mechanisms responsible for the development and progression of DN remain poorly understood. Activation of multiple signaling pathways and key transcription factors can lead to aberrant expression of DN-related pathologic genes in target renal cells. Increasing evidence suggests that epigenetic mechanisms in chromatin such as DNA methylation, histone acetylation, and methylation can influence the pathophysiology of DN and metabolic memory. Exciting researches from cell culture and experimental animals have shown that key histone methylation patterns and the related histone methyltransferases and histone demethylases can play important roles in the regulation of inflammatory and profibrotic genes in renal cells under diabetic conditions. Because histone methylation is dynamic and potentially reversible, it can provide a window of opportunity for the development of much-needed novel therapeutic potential for DN in the future. In this minireview, we discuss recent advances in the field of histone methylation and its roles in the pathogenesis and progression of DN.

## 1. Introduction

Diabetic nephropathy (DN) is a well-known microvascular complication of diabetes and the leading cause of end-stage renal disease (ESRD) all over the world which contributes greatly to morbidity, mortality, and most health care costs [1]. DN clinically manifests as initial increase in glomerular filtration (GFR), microalbuminuria, proteinuria, glomerulosclerosis, increased creatinine levels, and eventual decreased GFR [2–4]. Well-described characteristic histological and pathological changes of DN in glomerulus, tubulointerstitium, and vasculature are virtually indistinguishable in both type 1 and type 2 diabetes [5, 6]. Typical glomerular changes include mesangial cell proliferation and hypertrophy caused by excessive extracellular matrix (ECM) protein accumulation and glomerular basement membrane (GBM) thickness, podocyte loss, and foot process effacement, which eventually lead to nodular glomerular sclerosis known as Kimmelstiel-Wilson lesions [7–9]. Similar changes occur in the tubulointerstitium after glomerular changes, including tubular basement membrane (TBM) thickness, tubular hypertrophy, and interstitial fibrosis due to EMT process [10, 11]. Hyaline arteriolosclerosis is often prominent caused by endothelial dysfunction and inflammation [5, 9, 12].

All the cell types of kidney were involved in the pathogenesis of DN including mesangial cells (MC), endothelial cells (EC), glomerular podocytes, tubular epithelia, interstitial fibroblasts, vascular endothelia, and infiltrating monocytes/macrophages/lymphocytes due to hyperglycemia. Hyperglycemia and complex interactions between environmental and genetic factors are responsible for the development of DN [5, 13], which lead to a lot of intracellular events including increased flux of polyols and hexosamines, activation of reactive oxygen species (ROS), and advanced glycation end products (AGEs); activation of the PKC, renin-angiotensin system (RAS), transforming growth factor *β*-Smad-mitogen-activated protein kinase (TGF-*β*-Smad-MAPK), Janus kinase-signal transducer and activator of transcription (JAK-STAT) pathways, and G protein signaling; deregulated expression of cyclin kinases, and their inhibitors; and aberrant expression of ECM proteins, ECM-degrading enzymes, metalloproteinases, and their inhibitors [5, 14–23]. All the above factors can induce aberrant expression of profibrotic and proinflammatory cytokines, cell-cycle genes, and ECM genes involved in DN [24]. There is cross talk among the above different signaling pathways that can amplify the aberrant pathogenetic gene expression and lead to the progression of DN. Emerging evidences showed that microRNAs (miRNAs) are involved in the cross talk among RAS, AGE/RAGE, and ROS in the context of DN [25]. Despite current understanding of the mechanism of DN, still there are not enough therapeutic approaches in preventing the progression of DN to ESRD, suggesting that further mechanism and mediators should be investigated for DN.

Another potential reason for the long-term progression of diabetic complication in kidney could be a metabolic memory phenomenon, early exposure of the target cells to high glucose (HG), leading to persistence of its deleterious effects after effective glycemic control. This cellular memory phenomenon was revealed by large-scale multicenter clinical trials such as the Diabetes Control and Complications Trial (DCCT) and the follow-up observational Epidemiology of Diabetes Intervention and Complications (EDIC) of the same cohort examined the long-term benefits of intensive therapy following the blood glucose normalization in type 1 diabetes group as well as in experimental models, and in type 2 diabetes patients the similar phenomenon referred to as legacy effect from the large-scale United Kingdom Prospective Diabetes Study (UKPDS) was also identified. The consistency across these studies is striking: that early glycemic control significantly delays but does not totally block the onset and the progression of diabetic nephropathy in both type 1 and type 2 diabetes [26–28]. Similar results were reported in recent animal models (diabetic dogs or rats) [29–31] and cell culture models (vascular smooth muscle cells and ECs) [32, 33], but the molecular determinants of metabolic memory in renal cells remain poorly understood. Since metabolic memory is bad for the prevention and treatment of DN, exploring the mechanisms underlying metabolic memory seems important. In recent years, epigenetic regulatory mechanisms have been well studied. Waddington originally defined epigenetics mainly to describe changes during embryonic development as “the casual interactions between genes and their products which bring the phenotype into being” [34]. More recently it has been broaden as “the structural adaptation of chromosomal regions so as to register, signal, or perpetuate altered activity states” to explain alterations in the chromatin state and structure in response to various cues [9]. Gene regulation controlled by the epigenetic mechanisms can play key roles in various health problems (type 2 diabetes, metabolic memory, autoimmune diseases, cancer, and autism) and also in phenotypic differences between monozygotic twins [35].

Epigenetics is widely known as the regulation of gene expression without changes in DNA sequence, and epigenetic modification consists of classic epigenetic mark as DNA methylation and posttranslational modifications (PTMs) of histones. These traditional epigenetic mechanisms, along with noncoding RNA (ncRNA), can modulate gene expression in the cell type specific pattern [9, 36]. Changes in epigenetic states can cause gene deregulation and pathological outcomes and can be implicated in the pathogenesis of various kinds of diseases including DN, uncovering how environmental factors, diets, and physical activities influencing the epigenetic modification could provide new insights into the pathogenesis of DN and new diagnostic biomarkers as well as preventive and therapeutic approaches for early intervention [9]. In eukaryotes, the way to achieve the regulation of genetic information mentioned above should be via the package of chromatic DNA which is inside a higher-order structure made up of a series of subunits named nucleosome. Every nucleosome has the folding of 147-bp liner DNA (approximately 2 meters) around the octamers comprising two copies of core histone proteins H2A, H2B, H3, and H4 [37, 38]. Histone tails can undergo more than 60 different types of modification; dynamic chromatin structure is modulated by PTMs on histones including acetylation and methylation of lysine (K) and arginine (R), phosphorylation of serine (S) and threonine (T), ubiquitylation and sumoylation of lysines, and ribosylation [39, 40]. It is difficult to decode the specific PTMs at the level of single histones and single nucleosomes; mounting evidence suggests that histone modifications “communicate” and influence each other [38]. Recent advances in the high-throughput technologies have greatly helped in cataloguing the proteins modified by abovementioned PTMs [41]. In the recent 14 years research on histone methylation has been the most flourishing field of epigenetics [42]; it is usually considered a prevalent modification among core histone tails and is one of the most stable PTMs and could be key factors in diabetic complications, but few studies have been done in DN. Here, we summarize this emerging area of research related to abnormal epigenetic interactions in DN, particularly focusing on the histone lysine methylations and their roles in the development and progression of DN.

## 2. Histone Methylations in DN

Methylation of a lysine residue was first reported in the* flagellin* protein of* Salmonella typhimurium* in 1959 by Ambler and Rees [41, 48] and histone methylation was depicted for the first time in 1964 [49]. Histone can be mono-, di-, or trimethylated on lysine and arginine residues which can add another layer of complexity to the posttranslational status on the histone tails; the predominant methylated sites in histones H3 include lysines 4, 9, 27, 36, and 79, while lysine 20 is methylated in H4 [50]. Histone methylation is associated with gene repression or activation depending on which residue is modified; in general, methylation at H3K4, H3K36, and H3K79 correlates with gene transcription, while methylation at H3K9, H3K27, and H4K20 correlates with transcriptional repression [51]. Unlike the histone acetylation linked to an “open” chromatin state and to the activation of gene expression, histone methylations have more diverse results: they can be marks of the active, “poised,” and repressive states of chromatin that has resulted in more attention in the explosion of studies to investigate the biological and pathological functions in the prevalent diseases including DM and its consequent complications such as DN. Below, we focus on the less well-studied role of histone lysine methylation in the development and progression of DN by two respective fields: the active chromatin marks and the repressive chromatin marks.

### 2.1. Active Chromatin Marks

In general, H3K4me1/2/3, H3K36me2/3, and H3K79me2 are associated with transcriptionally active regions; genome-wide mapping of above histone methylation regions showed important roles in the islet-specific promoters and enhancers for the pathogenesis of diabetes [9, 52]. Increasing evidences suggest that histone methylation are involved in the regulation of extracellular matrix (ECM) and inflammatory genes in almost all kinds of renal cell types associated with the pathogenesis of DN; we will talk about the functions of H3Kme in DN in the order of cell culture model, animal model, and DN patients.

#### 2.1.1. H3K4 Methylation

Recent studies indicate that H3K4me1/2/3 are important for the deregulation of key genes in the pathogenesis of DN. Among lots of factors and signaling contributing to DN pathogenesis, TGF-*β* can play important role in the expression of fibrotic and ECM genes such as collagen 1*α*1 (*Col1*α*1*), plasminogen activator inhibitor 1 (*PAI-1*), and connective tissue growth factor (*CTGF*) in renal cells. A recent study examined histone H3K4me in rat mesangial cell (RMC) treated with HG and TGF-*β*. TGF-*β* can induce* Col1*α*1*,* PAI-1*,and* CTGF* gene upregulation, which was associated with increased levels of H3K4me marks (H3K4me1/2/3) activation at their respective promoters [43]. HG treatment on RMCs can lead to similar changes in histone H3K4me at ECM associated genes promoters also, but the effects on the H3Kme changes by HG can be blocked by a TGF-*β* specific antibody, which showed a key role of TGF-*β* in the HG-induced histone H3K4me [43].

In transient hyperglycemic cell culture models, endothelial cells were stimulated by HG for 16 hours and then remained in normal glucose concentration medium, pleiotropic transcription factor* NF-*κ*B* subunit* P65* displayed sustained activation, and level of H3K4me1, but not H3K4me2 or H3K4me3, in the proximal promoter region of the* p65* gene was increased [33, 53]; two* NF-*κ*B p65*-activated inflammatory genes* MCP-1* and* VCAM-1* were increased and remained elevated, and transient hyperglycemia-induced* p65* gene upregulation was prevented by overexpression of* UCP-1*,* MnSOD*, or* GLO1*. Aortic endothelial cells isolated from nondiabetic mice model show that transient hyperglycemia can induce increased H3K4me1 at the* p65* promoter and increase* p65* gene transcription [33]. These results prove that metabolic memory exists in the vascular dysfunction arising from hyperglycemic exposure due to H3K4me modification.

Evidences of infiltrating blood cells recruitment are dependent on the key proinflammatory pathways in diabetes and vascular complications [54]; in cultured human monocytes (THP-1) stimulated with high glucose, results from genome-wide location analyses with ChIP coupled with DNA microarrays (ChIP-on-chip) show that H3K4me2 are dynamically changed; similar changes can be seen in the monocytes from type 1 and type 2 diabetes, which can provide clues to the “metabolic memory” phenomenon [55] by such profiling approaches.

Evidence for histone methylation changes in DN also comes from studies in animal models. One report showed that in uninephrectomized db/db mice kidney H3K4me2 level was increased in accordance with glomerular cell proliferation, albuminuria, and glomerular rate (GFR) reduction, and* MCP-1/CCL2* antagonist treatment can prevent DN histopathological damage and H3K4me2 change in uninephrectomized db/db mice [56]. Results of a recent study from type 2 diabetes model db/db mice showed that, relative to db/+ mice, H3K4me1 level in glomeruli from db/db mice was increased in accordance with RNA polymerase II recruitment enhancement at the promoters of PAI-1 and RAGE (receptor for advanced glycation end products), whereas no differences were noted for H3K4me2/3. Emerging evidences have implicated that Losartan, an Ang II type 1-receptor blocker (ARB), can slow down the progression of DN. Losartan treatment for 10 weeks in db/db mice can ameliorate key factors of DN, and there was no change in the H3K4me1 recruitment at* PAI-1* and* RAGE* promoters, suggesting that it cannot reverse H3K4me changes observed in the db/db mice and accounting for the incomplete inhibitory effect of ARBs in DN patients [44]. Another study in db/db mice kidney showed that ER stress can trigger the expression of inflammatory gene* MCP-1* associated with increased H3K4me1 at* MCP-1* promoter mediated by XBPs-induced* SET7/9* elevation [57].

Another report demonstrated that in type 1 diabetes models OVE26 mice and STZ induced rat renal gene expression of* Cox2*,* S100A4/FSP1*, and vimentin in both,* MCP-1* only in mice was upregulated, associated with increased H3K4me2 levels [47]. Some other studies showed that H3K4me3 at proinflammatory genes (*MCP-1* and* TNF*-*α* ), profibrotic genes (*TGF*-*β*1 and* collagen III*), and histone-modifying enzyme (Set1 and BRG1) were increased in renal ischemia-reperfusion injury animal models [58, 59], which provide hints for the DN underlying mechanism study in the future. All these results from animal models have shown that histone methylation plays important roles in the progression of DN in fibrotic, inflammatory, and oxidant stress ways.

#### 2.1.2. H3K36 Methylation

H3K36me3 was a chromatin mark associated with transcriptional elongation [60, 61]. In the study of glomeruli from db/dbH_2_O mice compared with db/+H_2_O mice, levels of H3K36me3 were higher at* MCP-1* and* RAGE* loci, and similar result was seen at the* PAI-1* gene but shows no statistical difference, and Losartan treatment for 10 weeks can slightly decrease H3K36me3 levels at the* RAGE* and* PAI-1* loci but not at the* MCP-1* gene [44]. This can further imply the roles of H3K36me in the progression of DN.

#### 2.1.3. H3K79 Methylation

Unlike most of the methylated sites that are located in the histone H3 tail, H3K79 methylated site is located in the histone globular domain. Methylation of H3K79 is catalyzed by the disruptor of telomeric silencing proteins DOT1/DOT1L and plays an essential role in cell cycle regulation, embryonic development, DNA damage response, hematopoiesis, cardiac function, and the development of leukemia [51]. One report showed that dynamic regulation of H3K79me was involved in fluid reabsorption essential for blood pressure control and electrolyte homeostasis in kidney collecting ducts: decreased H3K79me at epithelial sodium channel promoter can lead to increased gene expression in response to aldosterone signaling since H3K79 hypermethylation was associated with gene repression [62–64]. A recent study shows that both mice model and DN patients can develop polyuria due to the upregulation of Aqp5, in which Aqp5 acted as an Aqp2 binding partner and regulator and can impair Aqp2 membrane localization, and decreased H3K79me2 may contribute to the changes in DN patients and in mouse cortical collecting duct M1 cells models [45]. The known results can lead to great interest in investigating how specific gene promoters H3K79me were altered in other renal cells under diabetic conditions.

### 2.2. Repressive Chromatin Marks

H3K9me2/3, H3K27me3, and H4K20me3 are generally associated with gene silence or repression, and we will talk about them one by one following the orders of cell culture model, animal model, and DN patients mentioned above.

#### 2.2.1. H3K9 Methylation

Several recent reports showed that histone methylation may be responsible for the “metabolic memory” phenomenon leading to long-term changes in diabetic complications including DN. High glucose can stimulate the decreased H3K9me3 levels at the promoters of key inflammatory genes (*IL-6*,* MCSF*, and* MCP-1*) associated with increased inflammatory genes expression in normal human vascular smooth muscle cells (VSMC), similar chromatin lysine methylation changes were demonstrated in db/db mice VSMC compared to nondiabetic control db/+ VSMC, and TNF-*α* induction can lead to sustained decreases of H3K9me3 at promoters in accordance with increased inflammatory genes expressions in db/db VSMC [46, 63].

In TGF-*β* and HG treated RMCs models, TGF-*β* and HG can result in similar changes: induced* Col1*α*1*,* PAI-1*, and* CTGF* genes upregulation were associated with reduced levels of repressive marks H3K9me2 and H3K9me3 at their respective promoters; TGF-*β* specific antibody can block high glucose-induced H3K9me reduction at promoters similar to the blockade of H3K4me levels mentioned above [43]. Another study result showed that short time high glucose stimuli can induce a sustained reduction of H3K9me2 and H3K9me3 level on the* NF-*κ*B p65 *promoter even after glucose concentration returned to normal, suggesting that changes in histone methylation were associated with and potentially could partly explain the phenomenon of  “metabolic memory” [53]. Dynamic change in H3K9me2 can be seen in both cultured monocytes (THP-1) treated with high glucose and isolated monocytes from diabetic patients with genome-wide location analyses with ChIP coupled with DNA microarrays (ChIP-on-chip) [55] and in lymphocytes from type 1 diabetic patients H3K9me2 levels linked to immune and inflammatory pathways associated with type 1 diabetes, and its complications including DN in a subset of genes are increased with ChIP-on-chip study versus healthy controls, suggesting that histone methylation is cell type specific and relatively stable regardless of age or gender [65], which open an access to understanding of the pathogenesis for the progression of DN [66].

There are also evidences of H3K9me changes in DN from db/db mice glomeruli. ChIP assays results show that H3K9me2 and H3K9me3 levels in db/dbH_2_O mice at both* PAI-1* and* RAGE* gene promoters were lower compared with db/+H_2_O group, which was inversely correlated with their upregulation, and Losartan treatment cannot reverse DN-related changes but has further lower levels of H3K9me2 and H3K9me3 at* PAI-1* and* RAGE* gene promoters [44]. All the results from experimental models supplement roles of H3K9me in the development of DN.

#### 2.2.2. H3K27 Methylation

H3K27me is a “mark” associated with gene repression [67, 68]. A study result of ChIP assays from type 2 diabetes animal model db/dbH_2_O mice shows that H3K27me3 levels at* RAGE* and* PAI-1* promoters were decreased compared with db/+H_2_O, and Losartan treatment had little or no effect on DN-related H3K27me3 levels at the above two genes promoters [44]. Another report showed that in OVE26 mice and STZ induced rat type 1 diabetes models renal gene expressions of* Cox2* and* MCP-1* only in mice were upregulated, associated with decreased H3K27me3 levels, which was accompanied by H3K27me3 demethylase KDM6A species-specific increases in mice but not in rats [47]. Overall results of H3K27me3 further imply the roles of histone methylation in DN.

#### 2.2.3. H4K20 Methylation

Methylation of histone H4 was firstly discovered almost half a century ago and the catalyzing enzymes were identified recently. Histone H4 methylation was mostly detected on lysine 20; H4K20me1 and H4K20me2 are involved in DNA replication and damage repair, whereas H4K20me3 is related with gene repression; H4K20me1 was only catalyzed by SET8 (also known as PR-SET7), where H4K20me2 and H4K20me3 were predominantly mediated by SUV4-20H1 and SUV4-20H2 [50]. In a STZ induced rat model, poor glucose control can lead to retinal key antioxidant gene mitochondrial superoxide dismutase (*SOD*) downregulation by increased promoter levels of H4K20me3 through increased corresponding methyltransferase SUV4-20H2 recruitment to* SOD* gene promoter, suggesting that diabetic retinopathy is related with* SOD* repression in retinal ECs [29]. Much more interests should be of in the study of H4K20me in the pathogenesis of DN.

## 3. HMTs and HDMs in DN

Histone methylation is generally considered to be relatively more stable and is mediated by histone methyltransferases (HMTs) and histone demethylases (HDMs), which increase the complexity of the histone methylation in the pathogenesis of diseases and diabetic complications. Usually histone H3 lysine 4 methylation (H3K4me) can be mediated by lots of HMTs such as SET1/COMPASS, MLL1-4 (mixed lineage leukemia 1–4), SMYD2/3 (SET and MYND domain 2/3), and SET7/9 [69–74] and is associated with gene activation. On the other hand, histone H3 lysine 9 methylation (H3K9me) can be mediated by SUV39H1/2 (suppressor of variegation 3–9 homolog 1/2), G9a, GLP (G9a-like protein), SETDB1/ESET (SET domain, bifurcated 1/ERG-associated protein with SET domain), and Eu-HMTase1 [69, 71, 72, 75, 76] and is associated with gene repression. In addition to these, there are several lysines, including histone H3 lysine 27 methylation (H3K27me) mediated by EZH2 [77], histone H3 lysine 36 methylation (H3K36me) mediated by Set2 [78], and histone H3 lysine 79 methylation (H3K79me) mediated by Dot1 [51] that can be methylated to various degrees leading to altered gene expression. Now it is known that even lysine methylation is one of the most stable epigenetic modifications; it can be reversible by histone demethylase. The first identified histone demethylase is lysine demethylase 1 (LSD1), which can specifically remove H3K4me and H3K9me marks [79, 80]. Recently, a lot of lysine demethylases have been identified with varying specificities for different histone lysine residues [71, 81–83] whose nomenclature has been changed to lysine demethylases (KDMs). Dynamic regulation of lysine methylation can play important roles in various diseases and can be key factors in diabetic complications including DN.

Suv39h1 is a known HMT mediating H3K9me3; its protein level was decreased, associated with increased inflammatory genes expression and decreased H3K9me3 levels at inflammatory gene promoters in db/db mice VSMC compared to db/+ VSMC; Suv39h1 overexpression in db/db VSMC can reverse partial diabetic phenotype; TNF-*α* can induce inflammatory genes expression with corresponding decreased Suv39h1 occupancy and H3K9me3 levels at promoters [46, 63], suggesting a new direction for demonstrating potential roles of chromatin histone methylation in DN.

HMT SET7/9 recruitment and H3K4me marks appear to be characteristic of the insulin gene promoter activation only in cells associated with insulin production such as *β* cells, non-*β* cells, and embryonic stem cells (ES) [63, 84, 85]. Another study in monocyte showed that SET7/9 knockdown can decrease H3K4me1 at* MCP-1* and* TNF*-*α* promoters with a corresponding decreased NF-*κ*B subunit occupancies at promoters, which suggest that knockdown of SET7/9 can attenuate TNF-*α* induced key inflammatory genes expression in an* NF*-*κ*B-dependent manner. To further verify the effect of histone methylation and HMTs/HDMs in “metabolic memory,” results from recent cell culture studies show that transient hyperglycemia can induce the increased recruitment of the identified histone methyltransferases (HMTs) SET7/9 and histone demethylase (HDMs) LSD1 to the* p65* promoter in aortic endothelial cells [33, 53], which can lead to better understanding of the basis and strategies to reduce the burden of diabetic complications such as DN.

Recent studies have shown that TGF-*β* stimulation in rat renal mesangial cells can increase SET7/9 gene expression and SET7/9 recruitment to the promoters of key fibrotic genes (including* PAI-1* and* CTGF*) linked to DN, which are associated with active H3K4me1 occupancy; high glucose stimulation can lead to similar change in RMCs; TGF-*β* specific antibody treatment can reverse HG induced gene expression and promoter histone methylation changes; these results highlight a key role of histone methylation and HMT SET7/9 in modulating renal gene expression leading to the pathogenesis of DN [43].

A recent study of QPCR arrays screen of 86 genes encoding epigenetic-modifying enzymes and followed by RT-qPCR validation in the glomeruli shows that H3K4 methyltransferases (Setd4 and Setd7), H3K36me3 methyltransferase (Setd2), and H3K9me3 demethylases (Jmjd2 family) were increased in db/dbH_2_O mice, which can be inhibited by Losartan treatment [44]. A study mentioned above shows that renal Aquaporin 2- (*Aqp2*-) expressing cells (DOT1L^AC⁡^) deficient mice can develop polyuria due to the upregulation of* Aqp5*, and decreased H3K79me2 caused by DOT1L deficiency may contribute to the changes in mouse cortical collecting duct M1 cells models [45]. Evidences are progressed in investigating roles of HMTs/HDMs in the pathogenesis of DN.

Overall, these results of HMTs, HDMs, and interactions between them in various experimental models have shown the respective roles in cells relevant to DN but not sufficient, and it is anticipated that further research in the field of HMTs and HDMs may lead to clear description of the pathogenesis of DN.

## 4. Conclusions and Perspectives

Experimental studies including cell and animal models as well as clinical studies have clearly revealed deleterious results of hyperglycemia and importance of good glucose control in preventing the onset or progression of DN, and less proper therapeutic approaches are demonstrated. Histone lysine methylation is a dynamic process which enables another layer of gene expression control so that genes can be turned off in a cell type specific manner in response to various signaling and environmental stimuli, which has been found to play important roles in gene deregulation associated with various diseases [86]. We summarized ever demonstrated histone lysine methylation results in DN experimental models and fewer results in DN patients, which can partly explain the pathogenesis of DN but are not sufficient; new more critical information for DN will be provided with the development of microarrays and massively parallel NGS platforms. We also list histone lysine methylation changes and sustained corresponding inflammatory genes expression in endothelial cells under transient high glucose condition [33]; histone lysine methylation has similar effect in VSMC derived aortas of db/db mice, which provide new insights into metabolic memory; thus, it is likely that similar histone methylation changes can also occur in cells such as renal mesangial cells, tubules, and podocytes that are involved in DN. The interaction between HMTs and HDMs mentioned above adds complexity in DN gene expressions through histone lysine methylations mechanism (Table 1) but is not sufficient to imply the whole pathogenesis of DN. Like HDACs inhibitors have been shown to be a novel class of therapeutic agents in diabetic kidney injury [87–89]; evidences showed that inhibitors of various HMTs could be new epigenetic therapy agents for cancers [77, 90], which is hoped to be a new therapeutic field for DN in the future. In addition to histone lysine methylation studies, advances in other mechanisms such as microRNA mediated mechanism need to be explored for preventing the progression of DN.

## Figures and Tables

**Table 1:** Histone lysine methylations and HMTs/HDMs in DN.

Lysine	State	HMTs	HDMs	Target renal loci	Effects in DN	References
H3K4	me1 me2 me3	SET7/9 MLL1-4 MLL1-4	LSD1 LSD1	RMCs, ECs, and mice glomeruli RMCs RMCs	Upregulate profibrotic gene and stimulate NF-*κ*B activated inflammatory genes	Sun et al. [43] El-Osta et al. [33] Reddy et al. [44]

H3K36	me3			Mice glomeruli	Stimulate profibrotic and proinflammatory genes expression	Reddy et al. [44]

H3K79		DOT1/DOT1L		Kidney collecting ducts	Develop polyuria	Wu et al. [45]

H3K9	me2	SUV39H1/2 G9a	LSD1	RMCs, monocyte/lymphocyte, and mice glomeruli	Repress inflammatory/profibrotic gene expression	Sun et al. [43] Villeneuve et al. [46] Reddy et al. [44]
	me3	SUV39H1		Human/mice VSMCs, RMCs, and mice glomeruli		

H3K27	me3	EZH2	KDM6A	Mice glomeruli	Repress profibrotic genes expression	Reddy et al. [44] Komers et al. [47]

H4K20	me1 me2 me3	SET8 SUV4-20H1/2 SUV4-20H1/2			Repress SOD expression	Zhong and Kowluru [29]
